# Elevated basal levels of circulating activated platelets predict ICU-acquired sepsis and mortality: a prospective study

**DOI:** 10.1186/cc14109

**Published:** 2015-03-16

**Authors:** N Layios

**Affiliations:** 1 CHU Sart Tilman, Liège, Belgium

## Introduction

Platelets are now considered to be immune and inflammatory agents as well as key cells in coagulation, and as such have been implicated in the pathophysiology of sepsis [1]. Thrombocytopenia is associated with sepsis severity and poor prognosis, and hyperactivated platelets probably contribute to microvascular thrombosis and organ failure. In the present study, we evaluated platelet activation markers as potential predictive markers of sepsis and of mortality among four commonly encountered populations of patients admitted to ICUs.

## Methods

Ninety-nine non-infected ICU patients were prospectively screened at day 1 (T1) and day 3 (T2) of admission after elective cardiac surgery, trauma, acute neurologic dysfunction or prolonged ventilation (>48 hours). A third sample was drawn when infection was diagnosed (Tx). We evaluated platelet activation by measuring the expression of P-selectin (CD62P) and fibrinogen binding on the cell surface before and after stimulation with major platelet agonists (ADP, collagen, and TRAP) through flow cytometry. Clinical scores were obtained at admission.

## Results

Patients who developed sepsis (*n *= 18) presented with significantly higher platelet fibrinogen binding at T1 compared with patients who did not get infected (basal: *P *= 0.0014, upon stimulation: *P *< 0.0035). At T1, ROC AUC for association of basal fibrinogen binding with the occurrence of sepsis was 0.79 (95% CI: 0.68 to 0.89). Elevated basal CD62P expression level was associated with increased 90-day mortality (*P *= 0.042, ROC AUC = 0.78 (0.64 to 0.88)). Kaplan-Meier survival curves illustrated that mortality was significantly higher after stratification based on T1 basal CD62P level (cutoff MFI>31.56, HR = 13.6, *P *= 8.23 × 10^-6^). Multivariate logistic regression analysis using clinical scores (SOFA, APACHE II, SAPS II, SAPS III) indicated that addition of CD62P level or of bound fibrinogen level significantly improved prediction of mortality (odds ratio 1.078, *P *= 0.003) and sepsis (odds ratio 1.033, *P *= 0.0012), respectively.

## Conclusion

Predisposition to severe infection in selected critically ill medico-surgical adults can be identified on day 1 of admission based on circulating basally activated platelets. Levels of activated platelets may add incremental prognostic information to clinical scoring.
