# Erratum: Elevated basal levels of circulating activated platelets predict ICU-acquired sepsis and mortality: a prospective study

**DOI:** 10.1186/s13054-015-1005-7

**Published:** 2015-08-26

**Authors:** N. Layios, C. Delierneux, A. Hego, J. Huart, A. Joly, P. Geurts, P. Damas, C. Lecut, A. Gothot, C. Oury

**Affiliations:** Department of General Intensive Care, University Hospital Centre of Liège, Domaine Sart-Tilman B35, 4000 Liège, Belgium; GIGA-Cardiovascular Sciences, Laboratory of Thrombosis and Hemostasis, University of Liège, Domaine Sart-Tilman B35, 4000 Liège, Belgium; CHU de Liège, Domaine Sart-Tilman B35, 4000 Liège, Belgium; Systems and Modeling, Department of Electrical Engineering and Computer Science and GIGA-R, University of Liège, Domaine Sart-Tilman B35, 4000 Liège, Belgium; Laboratory Hematology, University Hospital Centre of Liège, Liège, Belgium

## Erratum

After publication of our recent Poster presentation [1], we noticed that C. Delierneux, A. Hego, J Huart, A Joly, P Geurts, P Damas, C Lecut, A Gothot and C Oury had been inadvertently omitted as co-authors. The author list is now complete and the competing interests section modified accordingly.
